# Understanding the opaque-is-more bias and saturated-is-more bias for colormap data visualizations

**DOI:** 10.3758/s13414-025-03172-w

**Published:** 2026-02-23

**Authors:** Melissa A. Schoenlein, Mouloukou Sidibe, Karen B. Schloss

**Affiliations:** 1 Department of Psychology, High Point University, One University Parkway, High Point, NC 27268 USA; 2 Department of Psychology, University of Wisconsin-Madison, 1202 W. Johnson Street, Madison, WI 53706 USA; 3 Wisconsin Institute for Discovery, University of Wisconsin-Madison, 330 N. Orchard Street, Madison, WI 53715 USA; 4 Department of Sports, Exercise, and Performance Psychology, Barry University, NW 115th Street, Miami Shores, FL 33168 USA

**Keywords:** Information visualization, Color cognition, Visual reasoning

## Abstract

When interpreting data visualizations, people have expectations of how colors should map onto quantities. These expectations are constructed from multiple biases, including the dark-is-more bias (darker colors represent larger quantities) and the opaque-is-more bias (regions appearing more opaque represent larger quantities), among others. The extent to which any one bias influences interpretations of data visualizations depends on the degree to which that bias is applicable for a given visualization (applicability principle) and its relative weight in combination with other biases (combination principle). However, basic questions remain concerning the perceptual conditions necessary to activate such biases so they become applicable. For example, in previous studies of the opaque-is-more bias, the test stimuli appeared to vary in opacity because they were created by interpolating between a “base” color and a background color, which was lighter or darker than the base color. As such, opacity variation was confounded with large lightness variation. From prior work, it is unknown whether the opaque-is-more bias can be activated without substantial lightness variation. Here, we varied opacity by varying colormap saturation relative to the background while reducing lightness contrast (holding L* in CIELAB constant). We found that the opaque-is-more bias can indeed be activated without substantial lightness variation. In the process, we also found evidence for a new, “saturated-is-more bias,” leading to expectations that regions greater in saturation map to larger magnitudes. These findings extend knowledge of how people infer meaning from visual features and can translate to inform design of effective information visualizations.

**Supplementary Information:**

The online version contains supplementary material available at 10.3758/s13414-025-03172-w.

In visual communication, designers create information visualizations by encoding concepts in visual features, and observers interpret the meaning of those visual features to make sense of the visualization (see Schloss, 2025, for a review). For example, in Fig. 1, a designer created a choropleth map of carbon dioxide emissions from coal across the globe by encoding the per capita carbon dioxide emissions using variations of blue. To interpret the visualization, observers determine which countries are more/less responsible for coal-related carbon dioxide emissions by comparing the shades of blue across the map. Without even looking at the legend, most observers are likely to expect that darker blues represent greater carbon dioxide emissions (Cuff, 1973; McGranaghan, 1989; Schloss et al., 2019). This expectation, or *inferred mapping* between visual features and concepts, stems from multiple biases that work together in this figure—the dark-is-more bias leads to the expectation that darker colors map to larger magnitudes, and the opaque-is-more bias leads to the expectation that regions that appear more opaque (less “see-through”) map to larger magnitudes. In the legend in Fig. 1, the *encoded mapping* between colors and magnitude matches the inferred mapping, but when they mismatch, observers have more difficulty interpreting data visualizations (Schloss et al., 2019; Sibrel et al., 2020; Soto et al., 2023). Thus, a general goal of research on visual communication is to understand the factors that contribute to people’s inferred mappings as they strive to interpret information visualizations (Cleveland & McGill, 1986; Hegarty, 2011; Heider, 1972; Lin et al., 2013; Mukherjee et al., 2022; Norman, 2013; Schloss et al., 2018, 2019, 2021, 2023; Schoenlein et al., 2023; Sibrel et al., 2020; Tversky, 2011; Zimnicki et al., 2023).

**Fig. 1:**
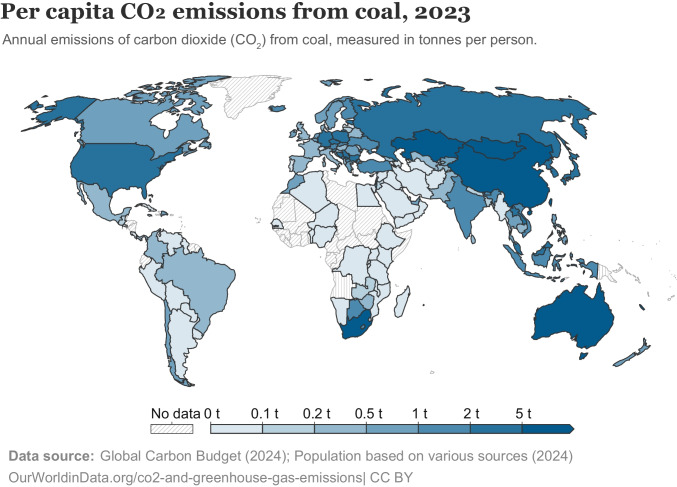
A choropleth map of per capita CO_2_ emissions from coal across the globe, in which darker and more saturated colors map to larger quantities (colors adapted to be consistent with opacity variation on white background)

Two key principles have been proposed to govern the way distinct factors contribute to inferred mappings: the *applicability principle* and the *combination principle* (Schloss et al., 2023). Here, we will explain these principles in the context of colormap data visualizations, in which gradations of color represent gradations of magnitude in a dataset (as in Fig. 1). The applicability principle states that a particular factor only influences inferred mappings if it is applicable to the visualization, given the perceptual properties of the visualization. For example, the dark-is-more bias is applicable when colormaps appear to vary in lightness, and the opaque-is-more bias is applicable when colormaps appear to vary in opacity. Colormaps appear to vary in opacity when the colors within the colormap are interpolated between a “base” color and the background color (e.g., value-by-alpha maps; Roth et al., 2010). The colormap data visualization can be thought of as a *heterogenous figure* that represents data magnitude with different degrees of opacity. This figure is placed on a *homogenous ground*, and the color appearance of the surface is determined by the opacity level of the figural region and the ground color below (Fig. 2, left).^1^ Such configurations are distinct from classic examples of perceptual transparency/translucency, in which a homogenous figure (i.e., a surface with a single level of translucency) is presented on a heterogenous background (e.g., Beck & Ivry, 1988; Khang & Zaidi, 2002; Metelli, 1974; Singh & Anderson, 2002) (Fig. 2, right).

**Fig. 2:**
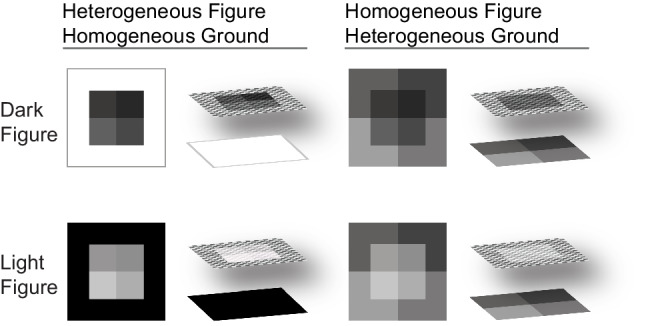
Examples of percepts of translucency achieved by varying opacity of a heterogeneous figure on a homogenous ground (left) or not varying opacity of a homogenous figure presented on a heterogenous ground (right). Figure from Schloss et al. (2019)

The *combination principle* states that when multiple factors are applicable, they combine to produce the inferred mapping for a given visualization (Schloss et al., 2023). These factors may work together or conflict. When factors conflict, they can cancel out or some factors can dominate others, depending on their relative strength (Bartel et al., 2021; Schloss et al., 2019; Schoenlein et al., 2023; Sibrel et al., 2020). For example, in Fig. 1 and Fig. 3A, the dark-is-more bias and opaque-is-more bias are both activated and they work together because on a light background, darker colors appear more opaque. However, when both biases are activated and the background is dark, as in Fig. 3B, the two biases conflict—lighter colors appear more opaque. Under such conflicts, the opaque-is-more bias tends to cancel out or override the dark-is-more bias, leading to inferences that lighter colors are mapped to larger magnitude (Schloss et al., 2019). The background color has little to no effect when colors in visualizations do not appear to vary in opacity (Bartel et al., 2021; McGranaghan, 1989; Schloss et al., 2019), indicating that the opaque-is-more bias is not merely a contrast bias (see Schloss et al., 2019, for a detailed discussion, and see Zimnicki et al., 2023, for potential exceptions). The existence of background effects on interpretations of data depicted in visualizations challenge the classic notion that the background serves no critical role in communicating information (though the original claim was made with respect to charts and graphs; Kosslyn, 1989).

**Fig. 3:**
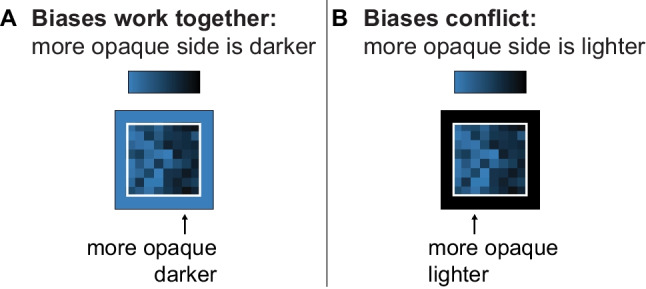
Colormaps from Schloss et al. (2019) in which both the opaque-is-more bias and dark-is-more bias are applicable and combine. **A.** The opaque-is-more bias and dark-is-more bias work together, such that they both suggest the same inferred mapping (dark-more). **B.** The opaque-is-more bias and dark-is-more bias conflict, such that the opaque-is-more bias suggests a light-more mapping, while the dark-is-more bias suggests a dark-more mapping

In the present study, we aimed to develop a deeper understanding of the perceptual conditions necessary to activate the opaque-is-more bias and elicit corresponding effects of background color on interpretations of colormap data visualizations. In previous studies that provided evidence for the opaque-is-more bias, opacity variation covaried with lightness variation (Bartel et al., 2021; Schloss et al., 2019). That is, opacity variation was achieved by interpolating between a light (or dark) color and a substantially darker (or lighter) background color, respectively, as illustrated in Fig. 3. As such, it is unknown whether the opaque-is-more bias can be activated in the absence of substantial lightness variation. Addressing this question is important as the field works towards producing a comprehensive model that can predict how people will interpret the meaning of colors across any data visualization by accounting for all the factors that contribute to inferred mappings. To effectively model the contribution of the opaque-is-more bias, it is necessary to understand the perceptual conditions in which it is applicable.

There exists evidence in the literature to support arguments for and against the possibility that the opaque-is-more bias can operate without strong lightness contrast. Evidence supporting the possibility comes from Ekroll et al. (2004), who found that percepts of translucency in 2D scenes can arise from isoluminant stimuli. Evidence against the possibility comes from studies on opacity/translucency perception in 3D objects (Fleming & Bülthoff, 2005; Motoyoshi, 2010). For example, most observers perceived cubes as translucent if they varied in lightness and had uniform saturation, but not if the cubes had uniform lightness and varied in saturation (Fleming & Bülthoff, 2005). Given that colormap displays can be considered as 2D scenes comprised of superimposed surfaces, Ekroll et al.‘s (2004) findings of the existence of translucency percepts in the absence of lightness variation may be more apt for the present study.

To disentangle opacity variation and lightness variation, we investigated whether the opaque-is-more bias could be activated when lightness contrast was reduced. To produce colormaps that varied in opacity with reduced lightness variation, we created color scales that interpolated between a base color and background color of equal lightness level (L* in CIELAB space). We say “reduced” lightness contrast (rather than zero lightness contrast) in this study because we controlled for lightness colorimetrically (constant L*) and presented the stimuli online using participants’ own devices (assuming an sRGB display). These viewing conditions approximate the viewing condition when observers examine information visualizations in the real world (e.g., on their computers, phones, TVs) and offer the degree of stimulus control one could achieve for real-world visualization (Gramazio et al., 2017; Stone et al., 2014; Szafir, 2017).^2^

Across all experiments, participants reported which side of the colormap corresponded to “more” (i.e., data of larger values) using only their inferred mappings (no legend or labels). In Experiments 1–2 (Fig. 4A), the color scales varied in saturation and the background either matched the desaturated (gray) or saturated (blue or green) endpoint of the scale. The saturated colors appeared more opaque on the gray background and the gray colors appeared more opaque on the saturated background (as verified in an additional experiment reported in the Supplementary Material; Fig. S2 and Table S2). These stimuli also enabled us to test for a potential saturated-is-more bias, leading to the inference that regions appearing more saturated map to larger quantities. In Experiment 3, we aimed to isolate the opaque-is-more bias from effects of lightness and saturation by testing color scales that varied in hue from green to blue (Fig. 4B). Greener regions should appear more opaque on the blue background, bluer regions should appear more opaque on the green background, and neither bluer nor greener regions should appear more opaque on the gray background. Finally, based on evidence for a saturated-is-more bias in Experiments 1 and 2, Experiment 4 aimed to isolate a saturated-is-more bias from effects of lightness and opacity (Fig. 4C). We tested green-gray and blue-gray color scales presented on the alternate hue background (blue or green, respectively), such that neither endpoint of the color scale should appear more opaque. The stimuli, data, and analysis code for all experiments can be found at OSF (https://osf.io/qv8tp/).

**Fig. 4:**
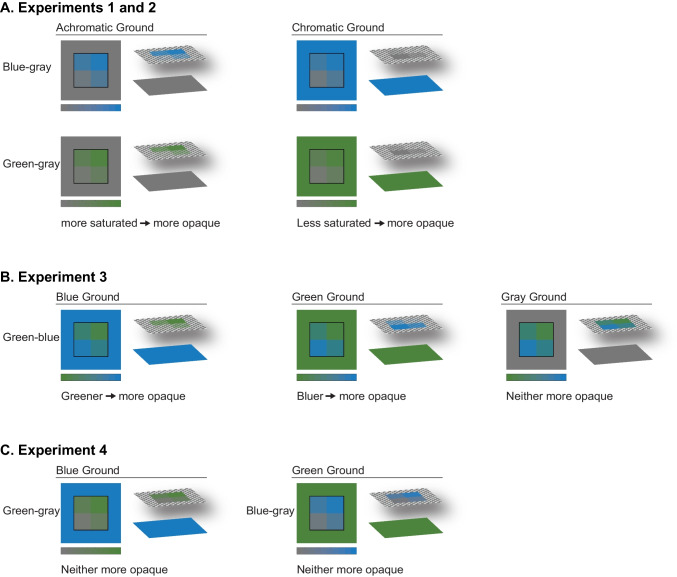
Schematic diagrams of the map stimuli placed on different backgrounds, with the color scale used to generate the maps illustrated below, and the stimuli split into component surfaces illustrated to the right. The lower layer represents the background and the upper layer represents the map, with a checkerboard used to indicate regions of the map that appear translucent. In Experiments 1–2 (**A**), blue-gray colormaps and green-gray colormaps appeared on gray or saturated backgrounds. In Experiment 3 (**B**) green–blue colormaps appeared on blue, green, and gray backgrounds. In Experiment 4 (**C**), green-gray colormaps and blue-gray colormaps appeared on blue and green backgrounds, respectively

## Experiment 1

**Fig. 5:**
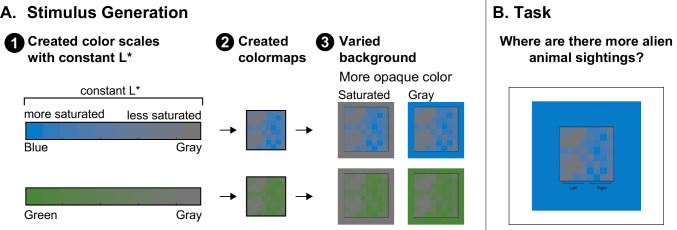
Stimulus generation and task in Experiment 1. **A.** Stimulus generation began with creating color scales that had a constant L* (L* = 50), then applying those color scales to underlying data to create colormaps, and then placing those colormaps on different backgrounds resulting in the different endpoint colors varying in opacity. **B.** Example trial in which participants indicate if they think the left or the right side of the colormap has more alien animal sightings

Experiment 1 tested if the opaque-is-more bias would activate for colormaps that varied in opacity by varying saturation relative to the background color and holding lightness (L*) constant. To create these stimuli, we set all colors in the display to have the same lightness value (CIELAB L* = 50) and varied the colors in terms of saturation using two color scales: blue-gray, and green-gray (Fig. 5). When these colormaps are placed on a gray background, the more saturated endpoint (blue or green) appears more opaque and when presented on a saturated background (blue or green, respectively), the desaturated endpoint appears more opaque (see Supplementary Fig. S2 and Table S2). Following Schloss et al. (2019), the participants were told the colormaps represented data about alien animal sightings in different regions of the planet Sparl. We used a fictitious domain to avoid prior knowledge about colors related to the dataset. During the task, participants indicated which side of the colormap (left/right) represented more sightings (Fig. 5B).

Although the primary focus of this experiment was the opaque-is-more bias, we also aimed to test for the possibility of a new, saturated-is-more bias. A saturated-is-more bias would lead to the inference that regions appearing more saturated map to larger quantities. Following the combination principle, the opaque-is-more and potential saturated-is-more bias would work together when the background is desaturated, leading to a strong inference that saturated regions map to larger quantities. However, these two biases would conflict when the colormap is presented on a saturated background. The saturated-is-more bias could reduce, or even override the effect of the opaque-is more bias on inferred mappings, leading participants to infer that the less opaque, more saturated region maps to larger quantities.

### Methods

#### Participants

We collected data from 102 participants recruited from Amazon Mechanical Turk (slots posted via CloudResearch; formerly TurkPrime; see Litman et al., 2017). Seven participants were excluded for atypical color vision, which was assessed at the end of the experiment using 11 digitally rendered Ishihara Plates and their answers to the following two questions: (1) “Do you have difficulty seeing colors or noticing differences between colors compared to the average person?”, and (2) “Do you consider yourself to be colorblind?” Participants were excluded from analyses if they answered incorrectly to three or more of the Ishihara plates and/or if they answered “yes” to either of the color vision questions. Based on self-reported responses to open-ended demographic questions, the remaining 95 participants had a mean age of 39 years (range: 20–70 years), with 58 men and 37 women (none reported additional genders). Race/ethnicity were reported as: nine African American, six Asian, four Black, one Hawaiian, four Hispanic, two Latinx, two Mixed/Biracial, one Romanian, and 67 White participants. In this and all subsequent experiments, the participants could self-report multiple races/ethnicities, so the total number of races/ethnicities can exceed the number of participants. Participants were told that the experiment was expected to last about 5 min and they would receive $0.60 for their participation. All participants of this experiment and the following experiments provided informed consent and the University of Wisconsin–Madison IRB approved the protocol. Each experiment tested a different set of participants.

Our sample size was determined from a power analysis using previous data collected from a similar task. The power analysis indicated that 20 participants per condition were needed for a power of .85 to detect a significant interaction of background color and lightness, which together contributed to the percept of opacity. We expected to exclude ~ 9% of participants for atypical color vision so we initially posted 100 time slots to account for these expected exclusions. After the initial 100 time slots, due to the automatic assignment of participants to conditions, participants starting the experiment but not completing it (*n* = 15), and color vision exclusions, one condition had only 18 participants. We posted additional time slots to reach *n* = 20 for this condition.

#### Design and displays

The experimental design included colormaps generated from two possible color scales (blue-gray or green-gray), placed on two possible backgrounds (green or blue to match the saturated endpoint of the colorscale or gray to match the desaturated endpoint of the colorscale), resulting in four between-subject conditions. Each participant judged 10 colormaps within their randomly assigned condition.

To create the experimental stimuli, we first selected a blue and green color of equal chroma and lightness to serve as the “saturated endpoints” and a gray of equal lightness to serve as the “desaturated endpoint” (see Table 3 in the Appendix for color coordinates). We chose these particular blue and green hues because they could be matched in CIELCh chroma (C*) and lightness (L*) at a high enough chroma level that both colors contrasted strongly with gray. Starting with these two base colors, we then created a blue-gray color scale and a green-gray color scale by linearly interpolating between each base color and gray (L* = 50) in CIELAB space (10 steps including the two end points of each scale), see Fig. 5A.

Second, we applied these color scales to 10 underlying data sets to create 10 colormap data visualizations for each color scale. The datasets (originally used in Schloss et al., 2019) were generated by sampling along an arctangent curve, such that the data values on one side of the curve were biased to be large and the other side were biased to be small (left/right balanced across the 10 datasets). As a result, for each color scale, half of the colormap data visualizations were more saturated on the right (as in Fig. 5) and the other half were more saturated on the left. Additional information about the underlying datasets can be found in the Supplementary Materials.

Third, we placed each colormap on a square background that matched either the saturated or gray endpoint of the color scale used to construct the colormap. Thus, we had four conditions: blue-gray on blue background, blue-gray on gray background, green-gray on green background, and green-gray on gray background. The colormap (4 cm $\times$ 4 cm) was centered on a background square (8 cm $\times$ 8 cm) which was centered on a white screen [RGB: (255, 255, 255)] (Fig. 5B). These dimensions describe the stimuli when presented on a 7.5 in. $\times$ 12 in. monitor with a 2,880 $\times$ 1,800 resolution. The dimensions could vary depending on the size of the participants’ monitors. Participants were asked at the end of the experiment what device they used to complete the experiment (Computer, Tablet, Phone, Other). All participants reported using computers. The stimuli were generated in MATLAB and the experiment program for this, and all subsequent experiments, were created using Javascript and the jsPsych packages (de Leeuw, 2015).

#### Procedure

The participants were instructed that they would see colormaps representing data collected by a scientist on the distant planet, Sparl. The scientist recorded alien animal sightings at different observation sites across the planet. Different parts of the sites varied in the amounts of alien animal sightings. In some cases, the left side of the site had more sightings. In some cases, the right side of the site had more sightings. Participants’ task was to look at the colormap and decide whether they thought there were more alien animal sightings on the left or right side of the site. Underneath the instructions text, the 10 colormaps from their condition were randomly displayed in a 5 column $\times$ 2 row grid.

After reading the instructions, participants pressed the “Next” button to begin the experimental trials. They then saw the 10 colormaps presented one at a time in a random order and indicated which side they thought had more alien animal sightings by pressing the left or right arrow key (Fig. 5B). Each trial was separated by a 500-ms intertrial interval with a “ + ” centered of the screen.

### Results and discussion

**Fig. 6:**
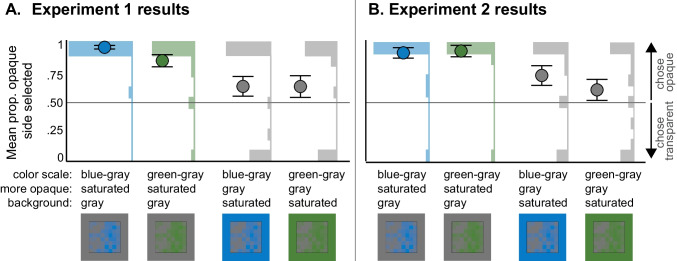
Results of (**A**) Experiment 1 and (**B**) Experiment 2. Plots show the mean proportion of times the opaque side was selected for each condition, in which the opaque side was either saturated or desaturated (gray). Error bars represent standard errors of the mean. Histograms show the number of participants who had a mean proportion of selecting the opaque side for each .1 interval

Figure 6A shows the mean proportion of times participants reported the more opaque side of the maps represented more alien animal sightings, depending on the condition in Experiment 1. These mean responses (represented as circular marks) are averaged across the 10 colormaps for each participant and then averaged over participants within each condition. Behind the circles are histograms of the individual participant responses, which ranged from 0 to 1 in 0.1 intervals given there were 10 trials. If the opaque-is-more bias were activated and it was the only factor governing inferred mappings in these displays, then participants in all four conditions would have responses near 1. However, responses appeared greater when the more opaque color was saturated rather than gray, which is consistent with a saturated-is-more bias working together with the opaque-is-more bias on the desaturated background and conflicting with the opaque-is-more bias on the saturated background.

To analyze this dataset, we first conducted a mixed effect logistic regression model using GLMER^3^ (Bates et al., 2011) to predict whether the more opaque side was selected on each trial (1 = yes, 0 = no) from a factor coding whether the more opaque side was saturated (.5) vs. desaturated (− .5), a factor coding whether the saturated side was green (.5) or blue (− .5), their interaction, and a by-subject random intercept. The results of the model are shown in Table 1. Only the intercept was significant, which indicates participants overall selected the more opaque side more often than chance, consistent with an opaque-is-more bias. According to this model, there was no significant effect of whether the opaque side was saturated or gray.

**Table 1:** Experiment 1 GLMER model predicting whether the more opaque side was selected from a factor coding whether the opaque side was saturated (.5) vs. gray (− .5) [*Saturation of Opaque Side*], a factor coding whether the saturated side of the colormap was green (.5) or blue (− .5) [*Saturated Hue*], their interaction, and a by-subject random intercept

	*Estimate*	*Std. Error*	*z value*	*p*	*OR*	*95% CI*
Intercept	11.758	1.780	6.604	***	127,830	[35153, 534401]
Saturation of Opaque Side	2.532	3.030	.836	.403	12.582	[.4398 4.040]
Saturated Hue	− 1.215	3.039	− .400	.689	.297	[.290, 2.569]
Saturation of Opaque Side*Saturated Hue	− 1.803	6.072	− .297	.767	.165	[.082, 7.439]

Odds ratio (*OR*) 95% confidence intervals [2.5%, 97.5%] calculated using boot method with 2,000 simulations (23 iterations failed to converge and were excluded)

We ran two follow-up GLMER models to test whether each of the two background conditions (whether the more opaque side was saturated versus desaturated) were both different than chance. In these models, we predicted whether the opaque side was selected more often than chance by coding for the overall intercept and by-subject random intercepts. The intercept was significant for both models, indicating the more opaque side was selected significantly above chance for when the opaque side was saturated (desaturated backgrounds) (β = 2.663, *SE* = .597, *z* = 4.4558, *p* < .001; odds ratio (*OR*) = 14.333, 95% CI [5.571, 9.069 × 10^9^]^4^), and when the opaque side was gray (saturated backgrounds) (β = .724, *SE* = .305, *z* = 2.376, *p* = .018; *OR* = 2.062, 95% CI [1.130, 3.90]). These models provide evidence that when the opaque-is-more bias conflicted with a possible saturated-is-more bias on saturated backgrounds, the saturated-is-more bias reduced the effect of the opaque-is-more bias, but did not entirely override it.

However, there are concerns that GLMER models fail to detect differences and can have substantial uncertainty in their estimates and effect sizes when large portions of the data are the same (i.e., nearly all participants of a given condition selecting the more opaque side; see histograms in Fig. 6A). Thus, we used another test that could be more sensitive to group differences for saturated versus desaturated background conditions. To prepare the data, we coded each participant according to whether they were an “opaque selector”—bars above .5 in Fig. 6A or a “transparent selector”—bars below .5 in Fig. 6A.^5^ For the purpose of this analysis, we excluded the three participants who had a mean proportion of selecting the opaque side of exactly .5 (chance). We then conducted a Fisher’s chi square performed on a contingency table to compare the frequencies of opaque vs. transparent selectors depending on whether the opaque side was saturated versus gray. Indeed, there were significantly more “opaque selectors” when the opaque side was saturated (desaturated background) than when the opaque side was gray (saturated backgrounds) (*p* < .001, *OR* = 20.940, 95% CI [2.959, 917.748]). These results are consistent with the possibility of a saturated-is-more bias, which worked together with the opaque-is-more bias on the desaturated background, but conflicted with the opaque-is-more bias on the saturated background, thereby reducing the probability that participants chose the opaque side when it was less saturated.

In summary, Experiment 1 provided evidence that the opaque-is-more bias was activated for colormaps that do not vary substantially in lightness, and provides preliminary evidence for a saturated-is-more bias.

## Experiment 2

The results of Experiment 1 suggest a potential saturated-is-more bias, but in Experiment 2 we considered an alternative explanation for the pattern of data. In Experiment 1, participants may have had an association between the concept of aliens and saturated colors (“direct” color–concept associations; Schoenlein et al., 2023), which could have driven them to infer that more alien sightings mapped to more saturated colors. To test this possibility, we modified the instructions so that instead of being told the colormaps represent alien animal sightings, participants were told that the data represent measurements, with no further specification of what the measurements meant. If the pattern of results are the same as Experiment 1, that would suggest that the inferred mappings in Experiment 1 were not due to direct associations with the concept represented by the colormap (i.e., alien sightings) but rather the saturated-is-more bias dampening the effect of  the opaque-is-more bias when they conflicted.

### Methods

#### Participants

We had the same target sample size of 20 valid datasets per condition as in Experiment 1. In total, we posted 103 new time slots to Amazon mTurk via CloudResearch (100 initial time slots and three additional to reach *n* = 20 per condition after exclusions). Of those time slots, 15 participants were excluded for atypical color vision, as assessed in Experiment 1. The remaining 88 participants self-reported a mean age of 39 years (range: 22–68 years), and gender as 42 men and 46 women (none reported additional genders). Race/ethnicity were reported as two African American, two American Indian/Native American, nine Asian, 12 Black, five Hispanic, two Latinx, one Mixed/Biracial, one Puerto Rican, and 56 White participants. Three of these participants reported using a tablet to complete the experiment, and the remaining participants reported using a computer.

#### Design, displays, and procedure

The design, displays, and procedure were identical to Experiment 1, except that the instructions indicated that the colormaps represented measurements collected in different parts of counties across the country. Participants were asked to indicate if they thought the measurements had larger numbers on the left or right side of the county.

### Results and discussion

The pattern of data in Experiment 2 (Fig. 6B) resemble the data in Experiment 1 (Fig. 6A) and the statistical tests revealed the same effects as in Experiment 1. In the GLMER analysis, only the intercept was significant (Table 2), indicating that participants selected the opaque side more often than chance (opaque-is-more bias). In the follow-up GLMER models testing whether the responses were different from chance for when the opaque side was saturated versus desaturated, the intercept was significant for both models, indicating the more opaque side was selected significantly above chance when the opaque side was saturated (desaturated background) (β = 3.0445, *SE* = .724, *z* = 4.207, *p* < .001; *OR* = 21, 95% CI [7.8, 3.588 × 10^9^]), and when the opaque side was gray (saturated background) (*β* = .659, SE = .318, z = 2.073, *p* = .038; OR = 1.933, 95% CI [1.095, 3.889]).

**Table 2:** Experiment 2 GLMER model predicting whether the more opaque side was selected from a factor coding whether the opaque side was saturated (.5) vs. gray (− .5) [*Saturation of Opaque Side*], a factor coding whether the saturated side of the colormap was green (.5) or blue (− .5) [*Saturated Hue*], their interaction, and a by-subject random intercept

	*Estimate*	*Std. Error*	*z value*	*p*	*OR*	*95% CI*
Intercept	9.559	1.470	6.505	***	14,170	[12225, 282841]
Saturation of Opaque Side	2.705	1.887	1.433	.152	14.951	[.506, 6.639]
Saturated Hue	− .591	1.857	− .318	.751	.554	[.244, 3.394]
Saturation of Opaque Side*Saturated Hue	1.608	3.725	.432	.666	4.992	[.103, 18.469]

Odds ratio (*OR*) 95% confidence intervals [2.5%, 97.5%] calculated using boot method with 2000 simulations (12 iterations failed to converge and were excluded)

The Fisher’s chi square test revealed there were significantly more opaque selectors when the opaque side was saturated (desaturated background) compared to when the opaque side was gray (saturated background) (*p* = .005, *OR* = 8.055, 95% CI [1.582, 80.202]), consistent with a saturated-is-more bias that reduced the probability of choosing the opaque-side when the opaque region was desaturated.

In summary, Experiment 2 replicated the pattern of results of Experiment 1 using a more domain-general cover story in the instructions to describe the data visualizations. These results provide further evidence that the opaque-is-more bias can be activated under minimal lightness variation, and suggest that the effects of saturation in Experiment 1 were likely due to a saturated-is-more bias and not due to the data representing alien animal sightings. We return to directly testing for the saturated-is-more bias in Experiment 4.

## Experiment 3

In Experiment 3, we tested if the opaque-is-more bias would activate for colormaps that varied in opacity due to variation in hue, without varying in lightness or saturation between the two endpoints of the color scale. To do so, we created a new color scale that varied from a saturated green endpoint to a saturated blue endpoint of equal lightness and saturation. We then presented colormaps generated using this color scale on either a blue, green, or gray background. The green side should appear more opaque on the blue background, the blue side should appear more opaque on the green background, and neither side should appear more opaque on the gray background. We used the same task and instructions as in Experiment 2.

### Methods

#### Participants

We collected data from 75 participants with a target sample size of 20 participants in each of the three conditions. One participant was excluded for not finishing and nine participants were excluded for atypical color vision, as assessed in Experiment 1. The remaining 65 participants self-reported a mean age of 36 years (range: 24–61 years), and gender as 21 men and 44 women (none reported additional genders). Race/ethnicity were reported as two African American, two American, four Asian, six Black, two Hispanic, one Latinx, and 48 White participants. Each of these participants reported using a computer to complete the experiment.

#### Design, displays, and procedure

**Fig. 7:**
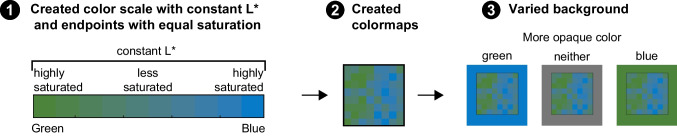
Experiment 3 stimuli creation. (**1**) A color scale varying from a saturated green to a saturated blue with uniform lightness (constant L*) was created. (**2**) The color scale was applied to the same underlying datasets as used in the previous experiments. (**3**) The colormaps were placed on different backgrounds, resulting in the more opaque side appearing as the green side (blue background), neither side (gray background), or blue side (green background)

All participants saw colormaps generated from the green–blue color scale and were randomly assigned to one of three background conditions (between-subject): blue, gray, or green (Fig. 7). The color coordinates for the base colors were the same as in Experiments 1–2 (Table A1). To create the appearance of opacity variation on the blue/green backgrounds, we created the green–blue color scale by interpolating between the blue and green colors in CIELAB space. Given the linear interpolation, the middle of the color scale was slightly less saturated than the endpoints. Importantly, both endpoints were equally saturated, so any activation of the saturated-is-more bias should be distributed across both sides of the colormaps and should not drive responses. Otherwise, the design and procedure were identical to Experiment 2.

### Results and discussion

**Fig. 8:**
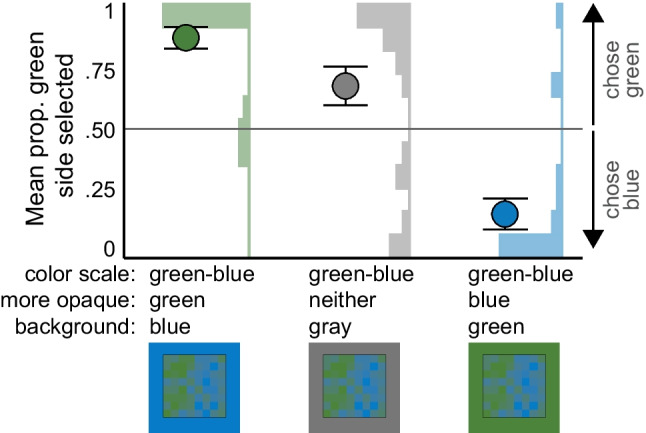
Experiment 3 results. Mean proportion the green side was selected for each background condition in which the more opaque color was either green, neither, or blue. Error bars represent standard errors of the mean. Histograms show the number of participants with a mean proportion of selecting the more green side at each .1 interval

Figure 8 shows the mean proportion of times participants chose the green side in each colormap for each background condition, and corresponding histograms at the subject level. Unlike Fig. 6, we did not plot the data according to the opaque side of the colormap because neither side should appear more opaque on the gray background. The opaque-is-more bias implies that participants would select the green side above chance on the blue background, below chance on the green background, and at chance on the gray background.

We used GLMER models to test whether each of the three background conditions were each different than chance. In these models, we predicted whether the green side was selected more often than chance by coding for the overall intercept and by-subject random intercepts. The intercept was significant for both models testing the saturated background conditions, indicating the greener side was selected significantly above chance for the blue background (β = 8.657, *SE* = 2.166, *z* = 3.9970, *p* < .001; *OR* = 5752.833, 95% CI [394.775, 606,228.3]), and significantly below chance for the green background (β =  − 7.227, *SE* = 2.510, *z* =  − 2.88, *p* = .004; *OR* = .001, 95% CI [0, .018]). These results demonstrate that the more opaque side was selected for each background. Surprisingly, the intercept was also significant for the gray backgrounds, indicating that participants were more likely to choose the green side on the gray background (β = 1.811, *SE* = .881, *z* = 2.056, *p* = .040; *OR* = 6.115, 95% CI [1.263, 3,012.055]). Moreover, comparing across conditions, Fisher’s chi square test indicated that participants were significantly less likely to choose the green side on the green than on the gray background (*p* < .001, *OR* = .089, 95% CI [.012, .445]) (consistent with the opaque-is-more bias), but the corresponding difference for the blue vs. gray background did not reach statistical significance (*p* = .132, *OR* = .233, 95% CI [.021, 1.470]).

This unexpected pattern of selecting green more often than blue for the gray background could potentially be explained through direct color–concept associations, but it is not obvious what those associations might be in this context. As described in Experiment 2, we aimed to avoid the influence of any potential direct color–concept associations by informing participants in the instructions that the maps represented “measurements” and the task was to indicate whether the measurements had larger numbers on the left or right side of the map, rather than indicating where there were more “alien animal sightings” as in Experiment 1. However, it is possible that the concepts of “measurements” or “larger numbers” are more associated with greenness than blueness and that is why participants were more likely to choose the greener region on the gray background. It is also possible that people have a hue-based relational bias, inferring that colors of certain hues represent the concepts “more” or “larger”. Future investigations may shed light on these possibilities.

Another factor that could play a role is the perceived relative distance between the colored regions and observer due to chromostereopsis. That is, for the majority of observers, surfaces that emit/reflect longer wavelength light appear closer than surfaces that emit/reflect shorter wavelength light (Faubert, 1995; Thompson et al., 1993; Vos, 2008), so the green side could have appeared closer than the blue side to most participants. However, such an explanation would also need to take into account Emmert’s law, in which regions appearing closer, despite being the same size and physical distance as others, should appear smaller (Emmert, 1881; Epstein et al., 1961). That is, if the greener side appeared closer due to chromostereopsis, it should also have appeared smaller. Follow-up studies are needed to address potential effects of region distance/size on inferred mappings.

In summary, the results for blue and green backgrounds in Experiment 3 suggest that the opaque-is-more bias can be activated by varying hue when both endpoints of the color scale are similar in lightness and saturation. The results for the gray background suggest that some factor drove participants to infer that the green side represented larger quantities and we highlighted some possible explanations that could be explored in future studies.

## Experiment 4

In Experiment 4 we further investigated the potential of a saturated-is-more bias by testing colormaps that varied substantially in saturation but not lightness or opacity. Participants saw the blue-gray and green-gray colormaps from Experiments 1–2, but to remove opacity variation, the colormaps were presented on the opposite saturated color background. That is, blue-gray maps appeared on a green background and green-gray maps appeared on a blue background. The task was the same as Experiments 2–3. If a saturated-is-more bias exists, participants should be more likely than chance to select the saturated side of the blue-gray and green–blue maps as representing greater magnitude.

### Methods

#### Participants

We collected data from 50 participants with a target sample size of 20 participants per condition. Four participants were excluded for atypical color vision, as assessed in Experiment 1. The remaining 46 participants self-reported a mean age of 40 years (range: 21–67 years) and gender as 22 men, one non-binary, and 23 women. Race/ethnicity were reported as one African American, one American, one American Indian/Native American, three Asian, six Black, one Chinese, four Hispanic, one Middle Eastern, one Mixed/Biracial, and 31 White participants. Each of these participants reported using a computer to complete the experiment.

#### Design, displays, and procedure

The design and procedure were identical to Experiment 2 with the following exception: participants were randomly assigned to one of two conditions: blue-gray colormaps presented on the green background or green-gray colormaps presented on the blue background.

### Results and discussion

**Fig. 9:**
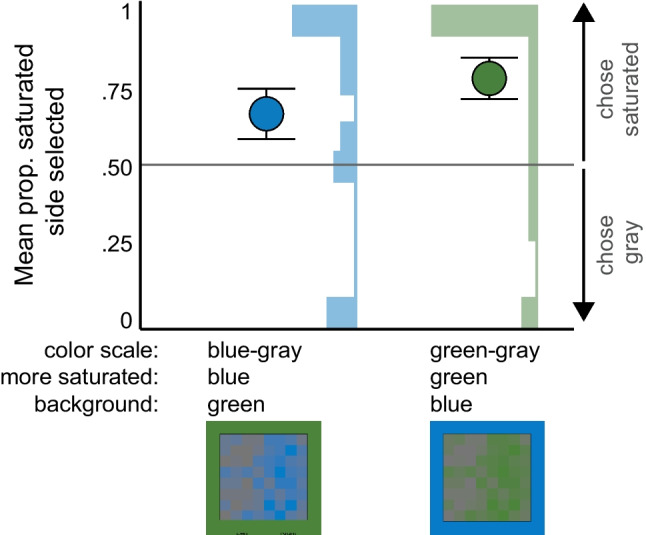
Experiment 4 results. Mean proportion of times the saturated side was selected for each background condition, in which the saturated side was either blue or green. Error bars represent standard errors of the mean. Histograms show the number of participants with a mean proportion of selecting the saturated side at each .1 interval

Figure 9 shows the mean proportion of times participants selected the more saturated side of the map for each condition and corresponding subject-level histograms. We used a GLMER model to predict whether participants selected the saturated side more often than chance by coding for the overall intercept and by-subject random intercepts. The intercept was significant (β = 3.974, *SE* = 1.732, *z* = 2.295, *p* = .022; *OR* = 53.205, 95% CI [11.428, 19,323.51]), indicating that participants selected the saturated side more often than chance. Fisher’s chi-square test indicates no significant difference in the number of participants from each condition^6^ selecting the saturated side versus the gray side (*p* = 1.00, *OR* = 1.260, 95% CI [.199, 8.003]). Thus, the results support the existence a saturated-is-more bias.

However, a potential alternative account should be considered. It is possible that participants perceived the colormaps not merely as two superimposed surfaces (heterogenous figure on homogenous ground; Fig. 2, left), but rather as three surfaces: (1) a heterogenous figure representing data (i.e., blue or green at varying opacity levels), placed on (2) a gray “data ground” that exists within a data region (i.e., inside black outline), placed on (3) an overall background (i.e., green or blue region outside of the black outline). If participants interpreted the colormaps in this way, it is possible their pattern of responses can be attributed to the opaque-is-more bias, rather than a saturated-is-more bias. Attempts to generate colormaps that eliminated this potential effect, while not appearing to vary in opacity and holding lightness constant proved to be challenging due to complex color interactions (e.g., color induction effects), so we leave this question open for future research.

In summary, the results of this experiment provide evidence for a saturated-is-more bias, which leads observers to infer that more saturated regions map to larger quantities. Although we cannot rule out alternative explanations in the present experiment, the results in Experiment 4, combined with Experiments 1 and 2 provide compelling evidence for the existence of a new, saturated-is-more bias.

## General discussion

The initial aim of this work was to investigate whether the opaque-is-more bias was applicable to colormap data visualizations with minimal variation in lightness. We had considered the possibility that substantial lightness variation may be needed to activate the opaque-is-more bias given previous evidence that lightness variation is important for the percept of translucency in 3D forms (Fleming & Bülthoff, 2005; Motoyoshi, 2010). However, aligning more with prior work on simple 2D scenes (Ekroll et al., 2004) we found that the opaque-is-more bias was activated despite reduced lightness contrast by varying saturation (Experiments 1–2) or hue (Experiment 3) in the colormap relative to the background color. The reliance on lightness variation for the appearance of translucency may be specific to 3D forms, as 3D form perception relies on lightness variation (e.g., shading) to discern an object’s shape, structure, and position relative to a light source (Ament et al., 2014; Ljung et al., 2016; Schott et al., 2009; Zhou et al., 2022). Without having to discern 3D form, the percept of opacity variation of heterogenous surfaces on homogenous grounds (Fig. 1) may depend less on lightness variation, and more on simple interpolations between surface color and background color (Roth et al., 2010). Open questions remain concerning how the opaque-is-more bias might operate in 3D information visualizations, in which shading is an important feature for communicating depth and curvature (e.g., volume renderings of anatomical structures in medical visualizations; Ament et al., 2014; Ljung et al., 2016; Schott et al., 2009; Zhou et al., 2022).

In this study, we also investigated the role of saturation on inferred mappings and found evidence for a new saturated-is-more bias (Experiments 1–2, 4). When saturation covaried with opacity, these biases worked together on a desaturated background and conflicted on the saturated background (Fig. 6). Under such conflicts, the saturated-is-more bias was strong enough to reduce the effects of the opaque-is-more bias (i.e., the probability of choosing the opaque region was closer to chance when the background was saturated) but not strong enough to override the opaque-is-more bias (i.e., responses were still above chance when the background was saturated). When we tried to dissociate saturation from opacity and lightness variation (Experiment 4), we found further evidence that participants inferred the more saturated regions mapped to larger magnitudes. However, we considered a potential alternative account that the gray in the colormaps was perceived as a “data ground” upon which a blue or green color scale varied in opacity to reveal the gray region below. If so, then the opaque-is-more bias could still be at play. Such an account implies three layers to the visualization: data, data ground within the potential data area, and background upon which the colormap is displayed. This potential three-layer interpretation prompts additional questions of whether these effects are primarily driven by lower level perceptual processing versus higher level knowledge about transparent surfaces. 

We note that the color scales in this experiment were designed to have limited variability to test hypotheses concerning the applicability of the opaque-is-more bias without large lightness variation and the existence of the saturated-is-more bias. However, we do not advocate for using these color scales in practice because lightness variation is important for revealing patterns of spatial variation in colormap data visualizations (Rogowitz & Kalvin, 2001; Rogowitz et al., 1996; Ware, 1988). Several resources and tools exist to aid in the process of selecting color scales that facilitate effective colormap design. For instance, ColorBrewer (Harrower & Brewer, 2003) provides several expert-made and tested color scales for sequential, diverging, and qualitative data. Color Crafter (Smart et al., 2020) is trained on color scales (also known as color ramps in Smart et al., 2020) designed by experts in the field, which allows it to generate new color scales for users that mimic best practices implemented by such experts. CCC-Tool (Nardini et al., 2019) allows for the automatic creation of color scales with high levels of customization. Each of these tools, among others, were created with the goal to provide support to designers for selecting color scales to represent a given data set, which takes into account factors such as uniformity, perceptual discriminability, and color deficiencies (see Bujack et al., 2017, for a review of these factors). Yet, our study with controlled color scales can help inform what factors influence people’s inference of how colors map to concepts in real-world visualizations.

Indeed, this study is part of a larger effort to develop a comprehensive understanding of all factors that contribute to inferred mappings for colormap data visualizations. This understanding will help to develop predictive models of colormap interpretation and to design visualizations that align with observer expectations. However, the task of designing visualizations that align with inferred mappings can quickly become complex when considering how multiple factors combine, especially when those factors conflict. Schoenlein et al. (2023) developed an approach for combining multiple factors to predict the participants’ ultimate inferred mapping. The approach is motivated by evidence that when people interpret color meaning in information visualizations, they evaluate the different possible color–concept assignments and infer the assignment that has the greatest overall “merit.” This process is called assignment inference (Schloss et al., 2018). Merit can be derived from multiple sources, including direct associations—the degree to which individual colors are associated with individual concepts (Mukherjee et al., 2022; Schloss et al., 2018, 2021; Schoenlein et al., 2023) and relational associations—the degree to which relations among colors correspond to relations among concepts (Schoenlein et al., 2023). For example, the dark-is-more bias is a relational association in that relatively darker colors are more associated with relatively larger quantities. Likewise, the opaque-is-more and saturated-is-more biases are relational associations, in that regions appearing relatively more opaque and more saturated map to relatively larger quantities, respectively. To combine multiple sources of merit, Schoenlein et al. (2023) empirically derived weights to place on each source, and then used the weighted sum to determine which color–concept assignment was optimal. They found that human judgments of which colors mapped to larger quantities in colormap data visualizations aligned with the model results.

Schoenlein et al. (2023) established this approach using only the dark-is-more bias and direct associations, but the present study suggests that the opaque-is-more and saturated-is-more biases can also be incorporated into this weighted sum by being treated as independent sources of merit. Future investigations will test this hypothesis with the goal of creating a comprehensive model that predicts people’s inferred mappings for information visualizations. This model can be used to design visualizations that match people’s expectations, thereby making visualizations easier to interpret.

In conclusion, our results suggest that there are many ways to activate the opaque-is-more bias without relying on strong lightness variation. We also found evidence for a new, saturated-is-more bias that leads to the inference that regions appearing more saturated represent larger data magnitudes. These findings contribute to the growing efforts to understand people’s expectations about the meanings of colors in colormap data visualizations, which can support better predictions of peoples’ expectations and inform the design of visualizations that facilitate visual communication.

## Electronic supplementary material

Below is the link to the electronic supplementary material.

Supplementary file1 (DOCX 821 kb)

## Data Availability

The stimuli, datasets, and analysis code all four experiments of this article are publicly available on OSF: https://osf.io/qv8tp/

## Appendix 1

**Table 3:** Experiments 1–4 color coordinates for the endpoint colors in each color scale. Coordinates in CIELAB, CIELCh, and sRGB space, using D65 as the white point and making standard assumptions about the monitors

Color	L	a	b	C	h	R	G	B
Blue	50	0	− 47	47	270	7	123	199
Green	50	− 33	33	47	135	76	132	60
Gray	50	0	0	0	0	119	119	119

## Footnotes

1 We note that when the ground is homogenous, figure heterogeneity is not necessarily required to activate the percept of translucency; homogenous figures can appear translucent on homogenous grounds (Ekroll et al., 2004).

2 We explored creating isoluminant stimuli for individual online observers using the minimally distinct border method (Boynton & Kaiser, 1968), but observers had difficulty providing systematic responses. Future investigations may explore producing isoluminant colormaps using the face-based methods in Kindlmann et al. (2002).

3 nlminbwrap optimizer used for this model and all others reported.

4 We report the odds ratios and corresponding 95% confidence intervals for each of our GLMER models, but these values should be interpreted with caution given the patterns of our data (i.e., participants are very consistent within themselves and there is little variability across participants) result in nearly all the variance in the model being captured by the random effects structure (conditional *R*^2^ = .992 vs. marginal *R*^2^ = .006). The coefficients of the fixed effects consequently become unstable in trying to explain the remaining minimal variance. Exponentiating these unstable coefficients results in large odds ratios with wide confidence intervals, which suggest high degrees of uncertainty in the estimates.

5 We note that although dichotomizing continuous variables can result in reduced power and loss of individual variability (Iacobucci et al., 2015), we chose to do so given the limitations of GLMER models and the strong evidence present in the plotted data suggesting participants’ responses were divided into two kinds of groups: those who adopted a strategy of selecting the opaque side versus those who adopted a strategy of selecting the transparent side.

6 Three participants from the blue-gray on green background condition and one participant from the green-gray on blue background condition were excluded from the Fisher’s test given they had a .5 proportion of selecting the more saturated side.

## Declarations

### Open practices: availability of data, code, and materials

None of the experiments were preregistered. The stimuli, datasets, and analysis code supporting the conclusions for all four experiments of this article are publicly available on OSF: https://osf.io/qv8tp/ [10.17605/OSF.IO/QV8TP].

### Conference presentation

A subset of these findings were presented at the 24th Annual meeting of the Vision Sciences Society in May 2024 held in St. Pete Beach, FL.
