# Supplementary material for: Understanding the opaque-is-more bias and saturated-is-more bias for colormap data visualizations
Source: Atten Percept Psychophys. 2026 Feb 23;88(3):69. doi: 10.3758/s13414-025-03172-w (PMC12929228; doi:10.3758/s13414-025-03172-w)
Supplement: Supplementary file 1 — Supplementary file1 (DOCX 821 kb) [file 13414_2025_3172_MOESM1_ESM.docx]

**Supplemental Materials**

**Isolating the opaque-is-more bias for colormap data visualizations**

Melissa A. Schoenlein, Mouloukou Sibide, Karen B. Schloss

**Experiment S1: Assessing opacity variation in colormap data visualizations**

This experiment tested whether the colormaps used Experiments 1 and 2 appeared to vary in opacity, and if there were any differences in apparent opacity variation between the colormaps from the different hue-background conditions. Participants saw the same displays as in Experiments 1 and 2 but their task was to indicate which side of the colormap was more “see-through.”

**Methods**

***Participants***

We collected data from 100 participants with a target sample size of 20 participants in each of the four hue-background conditions. Three participants were excluded for not finishing the experiment and 11 were excluded for atypical color vision, as assessed in Experiment 1. The remaining 86 participants self-reported a mean age of 40 years (range: 23 -77 years) and gender as 52 men, 33 women, and 1 no report. Race/ethnicity were reported as 2 African American, 6 Asian, 3 Black, 2 Hispanic, 1 Indian, 2 Latinx, 1 Middle Eastern, 2 Mixed/Biracial, 1 Mexican, 1 Slavic, and 66 White participants. Each of these participants reported using a computer to complete the experiment.

***Design, displays, and procedure***

The design, displays, and procedure were identical to Experiments 1 and 2, except that participants were asked to report which side of the colormap they thought appeared more “see-through.” We used this phrasing because it seemed easier for participants to understand the task when described in terms of transparency (i.e., see-through) rather than opacity. To further help ensure participants understood the task, we added an additional instructions page that provided a description of “see-through,” along with the example images in Figure S1, which featured colors varying in opacity overlaid on different background scenes (see Table S1 for color coordinates).


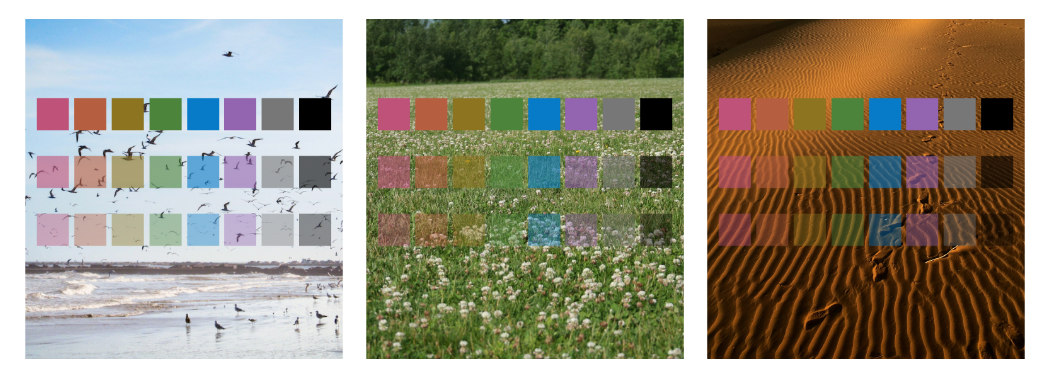


**Figure S1.** Example images shown during the instructions for Experiment S1 to demonstrate “see-through”. The following text appeared with the images: “To further explain what we mean by ‘see-through,’ below are three photographs with three rows of colored squares overlaid on each photograph. The rows of colored squares vary in how easy it appears to see through them: some rows appear more see-through (less opaque) than others. Please keep this idea in mind during the experiment. As a reminder, your task is to judge which side of the colormap appears MORE see-through.”

**Table S1.** Color coordinates for the colored squares presented in the instructions on opacity variation. Coordinates in CIELAB, CIELCh, and sRGB space, using D65 as the white point and making standard assumptions about the monitors.

| Color | L | a | b | C | h | R | G | B |
| --- | --- | --- | --- | --- | --- | --- | --- | --- |
| Red | 50 | 47 | 0 | 47 | 0 | 189.909 | 82.496 | 120.603 |
| Orange | 50 | 33 | 33 | 46.669 | 45 | 182.314 | 94.296 | 64.365 |
| Yellow | 50 | 0 | 47 | 47 | 90 | 140.414 | 116.892 | 32.782 |
| Green | 50 | -33 | 33 | 46.669 | 135 | 76.367 | 132.148 | 59.898 |
| Blue | 50 | 0 | -47 | 47 | 270 | 6.888 | 123.373 | 198.880 |
| Purple | 50 | 33 | -33 | 46.669 | 315 | 146.763 | 100.053 | 175.444 |
| Gray | 50 | 0 | 0 | 0 | 0 | 118.913 | 118.913 | 118.913 |
| Black | 0 | 0 | 0 | 0 | 0 | 0 | 0 | 0 |

**Results and Discussion**

Figure S2 shows the mean proportion of times participants chose the more opaque side as appearing more opaque. Given participants were asked to select the more see-through (less opaque) side, we inferred that the side not selected was perceived as less see-through (more opaque). We discuss the results and plot the data in terms of responses aligning with participants inferring the more opaque side (according to our definition of opaque) appeared as more opaque than the other side.


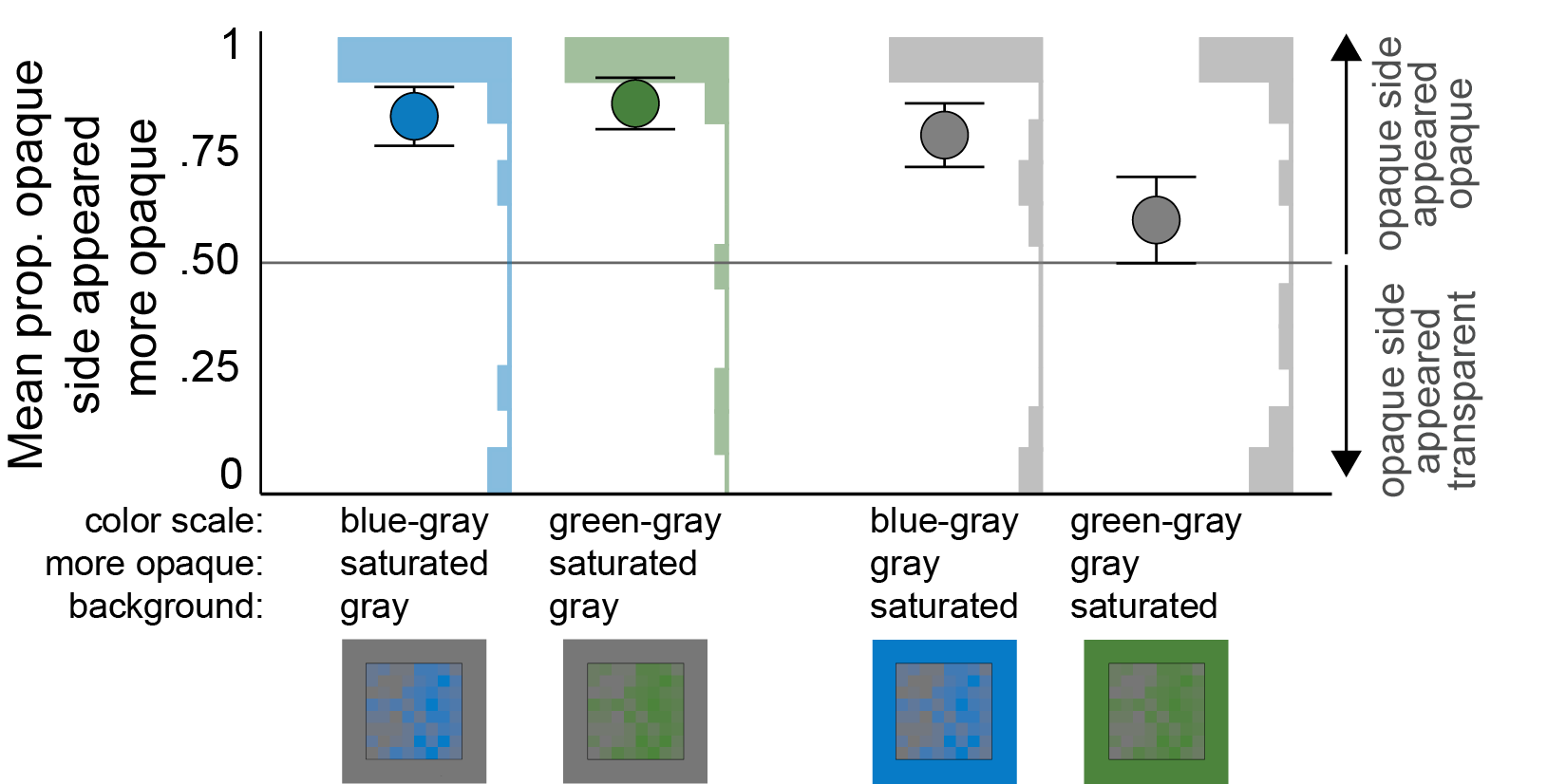


**Figure S2.** Results of Experiment S1. Plots show the mean proportion of times the opaque side was selected as appearing more opaque for each condition, in which the more opaque side was either saturated or gray. Error bars represent standard errors of the mean. Histograms show the number of participants with a mean proportion of the opaque side appearing more opaque at each .1 interval.

We used a Generalized Linear Mixed-Effect Regression model (GLMER)^[[1]](#footnote-1)^ to predict whether the more opaque side appeared more opaque (1 = no, 0 = yes; reverse coded from original task of selecting more see-through side) from a factor coding whether the more opaque side was saturated (.5) vs. desaturated (-.5), a factor coding whether the saturated side was green (.5) or blue (-.5), their interaction, and a by-subject random intercept. Only the intercept was significant, which indicates participants selected the more transparent side (less opaque) more often than chance as the side appearing more see-through. It follows, participants viewed the more opaque side more often than chance as the side appearing more opaque (Table S2).

**Table S2.** Experiment S1 GLMER model predicting whether the more opaque side was selected from a factor coding whether the more opaque side was saturated (.5) vs. gray (-.5) [*Saturation of Opaque Side*], a factor coding whether the saturated side of the colormap was green (.5) or blue (-.5) [*Saturated Hue*], their interaction, and a by-subject random intercept. Odds ratio (OR) 95% confidence intervals [2.5%, 97.5%] calculated using boot method with 2000 simulations (35 iterations failed to converge and were excluded).

|  | *Estimate* | *Std. Error* | *z value* | *p* | *OR* | *95% CI* |
| --- | --- | --- | --- | --- | --- | --- |
| Intercept | -8.540 | 1.351 | -6.320 | *** | .000 | [.000, .000] |
| Saturation of Opaque Side | -1.566 | 1.563 | -1.002 | .316 | .209 | [.166, 2.471] |
| Saturated Hue | .843 | 1.552 | .543 | .587 | 2.322 | [.333, 4.740] |
| Saturation of Opaque Side * Saturated Hue | -2.388 | 3.101 | -.770 | .441 | .092 | [.036, 7.666] |

We ran two follow-up GLMER models to test whether each background condition (saturated versus desaturated) were different than chance. In these models, we predicted whether the opaque side was selected from the intercept and a by-subject random intercept. The intercept was significant for both models, indicating the more opaque side was selected significantly above chance as appearing more opaque when it appeared saturated on gray backgrounds (*β* = -2.054, SE = .0475, z = -4.324, *p* < .001; OR = .128, 95% CI [.023, .294]), and when it appeared gray on saturated backgrounds (*β* = -.916, SE = .342, z = -2.681, *p* = .007; OR = .40, 95% CI [.167, .750]). Fisher’s chi square test also indicated no difference in the number of “opaque side selectors” versus “non-opaque side selectors” (*p* = .103, OR = .375, 95% CI [.092, 1.325])^[[2]](#footnote-2)^. Together, these data demonstrate two important results: (1) the colormaps used in Experiments 1 and 2 did appear to vary in opacity, and (2) there were no significant differences in perceived opacity depending on whether the opaque side was saturated (gray background) or desaturated (saturated background). These results help demonstrate the effects found in Experiments 1 and 2 are not simply due to differences in perceived opacity across the colormaps.

**Underlying data distribution for generating colormaps**

The following description of the process for generating the colormaps, including Fig. S.3, is reproduced directly from pp. 813-814 in Schloss et al. (2019):

The data used to generate the colormaps were sampled from an arctangent curve with added normally-sampled noise (Fig. S.1). To generate the data for each row of the colormap, we discretized the arc tangent curve into eight bins, corresponding to the eight columns in the colormap display. We centered the arctangent curve between the fourth and fifth bins, such that half of the display was biased to have larger values than the other half. We then perturbed each arctangent value by sampling from a normal distribution with the mean equal to the arctangent value and the standard deviation equal to 0.25. When the values fell outside the [0,1] interval, we re-sampled until they were all within the correct range. For half of the datasets, the arctangent curve was oriented as shown in Fig. S.1, and for the other half, it was left/right reversed. This enabled a left/right balance of the darker region (i.e., half of the colormaps contained the darker region on the left and the other half contained the darker region on the right).


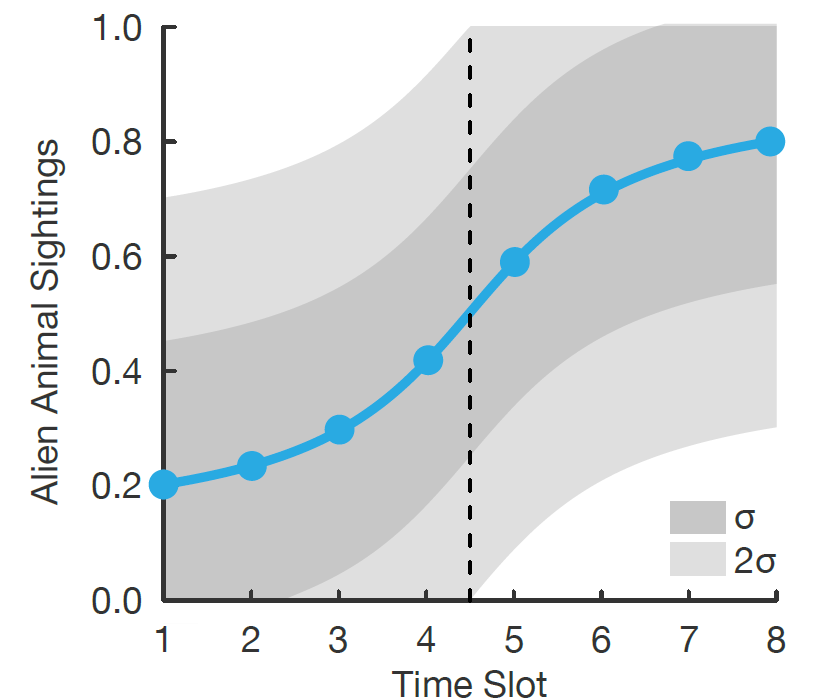


**Fig. S.3.** Distribution used to sample values at each time point to generate the data used to construct the colormap images. Figure reproduced from Figure 4 in Schloss et al. (2019).

1. nlminbwrap optimizer used for this model and all others reported. [↑](#footnote-ref-1)
2. One participant was excluded from the Fisher’s test given they had a mean proportion of exactly .5. [↑](#footnote-ref-2)
